# EpiRank: Modeling Bidirectional Disease Spread in Asymmetric Commuting Networks

**DOI:** 10.1038/s41598-019-41719-8

**Published:** 2019-04-01

**Authors:** Chung-Yuan Huang, Wei-Chien-Benny Chin, Tzai-Hung Wen, Yu-Hsiang Fu, Yu-Shiuan Tsai

**Affiliations:** 1 Department of Computer Science and Information Engineering, School of Electrical and Computer Engineering, College of Engineering, Chang Gung University, Taoyuan City, 33302 Taiwan; 2 Department of Geography, National Taiwan University, Taipei City, 10617 Taiwan; 3 Department of Computer Science, National Chiao Tung University, Hsinchu, 30010 Taiwan; 4 Department of Computer Science and Engineering, National Taiwan Ocean University, Keelung City, 20224 Taiwan

## Abstract

Commuting network flows are generally asymmetrical, with commuting behaviors bi-directionally balanced between home and work locations, and with weekday commutes providing many opportunities for the spread of infectious diseases via direct and indirect physical contact. The authors use a Markov chain model and PageRank-like algorithm to construct a novel algorithm called EpiRank to measure infection risk in a spatially confined commuting network on Taiwan island. Data from the country’s 2000 census were used to map epidemic risk distribution as a commuting network function. A daytime parameter was used to integrate forward and backward movement in order to analyze daily commuting patterns. EpiRank algorithm results were tested by comparing calculations with actual disease distributions for the 2009 H1N1 influenza outbreak and enterovirus cases between 2000 and 2008. Results suggest that the bidirectional movement model outperformed models that considered forward or backward direction only in terms of capturing spatial epidemic risk distribution. EpiRank also outperformed models based on network indexes such as PageRank and HITS. According to a sensitivity analysis of the daytime parameter, the backward movement effect is more important than the forward movement effect for understanding a commuting network’s disease diffusion structure. Our evidence supports the use of EpiRank as an alternative network measure for analyzing disease diffusion in a commuting network.

## Introduction

In light of the presence of network structures in most transmission processes^1^, topological network structures have utility for understanding the spread of messages^2–4^, diseases^5–9^, computer viruses^10,11^, innovative ideas^12,13^, human movement^14,15^ and rumors^16,17^, among other ideas and objects. Researchers have used social networks to study the contagious nature of obesity and emotion cognition^18–20^, as well as the spread of violence and wars via location network structures^21,22^. Some of the networks underlying these transmission examples are formed by human interaction, others by computer or mobile device connections, and still others by spatial links. Despite differences in physical meaning and mechanisms, these networks share the features of nodes representing transmission endpoints and links representing intersections where transmission occurs. Accordingly, transmission examples can be analyzed by conceptualizing endpoints and connections within the topological structure of a network.

Social scientists have utilized network topologies to model human connectivity in the form of social relationships, disease networks, and many other types of systems and complexes, using combinations of real world data and topological structure observations to analyze interactions between individuals and transmission characteristics^2,23^. Topological structure is now considered a key feature in the understanding of social network transmission events, especially differences in interaction behaviors based on different node and link types^24^. However, node interactions are subject to oversimplification as a binary variable (i.e., two nodes either do or don’t interact) at the expense of inspecting differences in quantity, interaction strength, and node influence. Clearly, having detailed information on nodes and interactions is central to understanding transmission processes in networks.

Since direct and indirect forms of physical interaction (e.g., coughing, sneezing) can transmit influenza viruses and other diseases, they are considered key components in both novel disease and seasonal influenza outbreaks^6,25^. Traditional diffusion models such as SIR are used to evaluate epidemic status factors such as infection threshold, infected and potentially infected individuals, and mortality rate, but they are not useful for addressing underlying human interaction in terms of differences in the ability to infect others. Accordingly, SIR-like mathematical models can help identify temporal development trends but not the effects of human movement, therefore they cannot be used by local or national health authorities to make disease control decisions based on spatial considerations. This is an important shortcoming because understanding human movement is key to controlling the spread of infectious diseases.

In spatial epidemiology studies, human movement is considered key to understanding disease spread and diffusion^26,27^. Grais *et al*.^28^ and Hufnagel *et al*.^29^ have used commuting flow to construct network structures for analyzing the influences of human movement on infectious disease diffusion, based on the belief that human movement and interaction (especially direct or indirect physical contact) support the spread of disease pathogens. Another way of describing pathogen movement is as a side effect of human movement, which allows for a social network perspective^30,31^ in which a transportation or commuting network serves as the key movement capturing factor^28^. Among infectious diseases, flu viruses (caused by droplets and physical contact) and enteroviruses (physical contact) have been shown to be strongly affected by transportation networks^32^.

**Figure 1:**
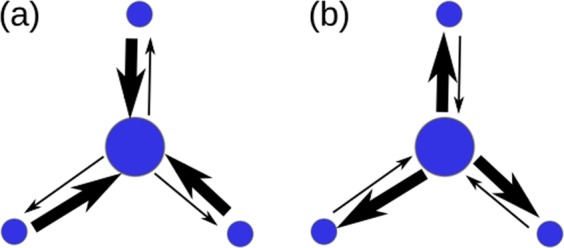
Illustration of a bidirectional transitive effect for two directed links. Nodes represent townships. (**a**) During morning hours, the majority of movement occurs from satellite areas (small nodes) to city centers (large nodes), with much less movement from city centers to satellite areas. (**b**) Similar flows move in opposite directions during evening hours.

Past human movement and disease diffusion process studies have generally focused on movement in one direction, usually from homes to workplaces or schools^33–35^. However, individuals in commuting networks move bi-directionally between their homes, offices, and schools on a daily basis. As shown in Fig. 1, disease transmission can also occur during movement to satellite locations during non-work hours. Accordingly, even though the number of individuals moving from core to satellite areas may be small, the number of individuals moving from satellite to core areas can be high. Such individuals may become infected while working in a core area and carry disease pathogens to satellite areas. In this study we will refer to these movements as “forward flow” (movement from homes to workplaces or schools) (Fig. 1a) and “backward flow” (movements toward residences) (Fig. 1b)^36,37^.

The use of bidirectional movement for analytical purposes differs from the conventional use of two separate links moving in opposite directions, which indicate travel from places of origin to other locations for work or school—that is, both represent forward flow (Fig. 1a). In such scenarios, weights and flows in different directions can be considered as independent or asymmetric. Referring to the Fig. 1a example, large numbers of individuals move toward central areas (heavy arrows) and much smaller numbers move toward satellite areas (light arrows); the evening pattern is shown in Fig. 1b. The two figures are representative of a conventional daily commuting network cycle based on a sense of bidirectionality that is key to modelling disease diffusion patterns.

Our study goals are to model the network transitive effect of this bidirectional movement and to design an algorithm for measuring the associated epidemic risk, using a Markov chain model to capture and measure the transitive effect. The PageRank algorithm^38^ was modified to create a new algorithm called EpiRank to capture bidirectional movement. PageRank is the most popular algorithm using the Markov chain model in transitive effect studies^38^. Spatial network researchers have tried to capture transitive patterns in human flow networks^14,39,40^, but to our knowledge none of them have included the concept of bidirectional movement in their calculations.

For this study we used two infectious diseases spread by droplets and physical contact (the H1N1 flu virus and Type 71 enterovirus, hereafter referred to as “flu” and “EV”) to test the ability of EpiRank to capture the influence of a bidirectional commuting pattern on disease dissemination in a commuting network in Taiwan. Commuting flows across township boundaries were used to construct the EpiRank origin-destination (OD) matrix. Since disease control operations generally use administrative boundaries as spatial units, our EpiRank analysis is based on commuting movement between townships.

## Methods

### Commuting data

Taiwan census data for year 2000 contains information on the residential and workplace townships of all surveyed individuals^41^. Assuming that commuting behaviors did not change dramatically during the ensuing decade, we used the 2000 census to extract commuting patterns on the main island of Taiwan. Spatial data covering 353 townships were used to construct a daily flow network and to create an asymmetrical square OD matrix consisting of 353 origin and 353 destination townships. Of the 21,335,199 people residing in these townships, 3,906,663 (18.31%) were identified as inter-township commuters. Since repetitive daily commuting movements reveal the basic flows of movement patterns, their influences on disease diffusion are relatively stable compared to the movement of individuals for other purposes.

**Figure 2:**
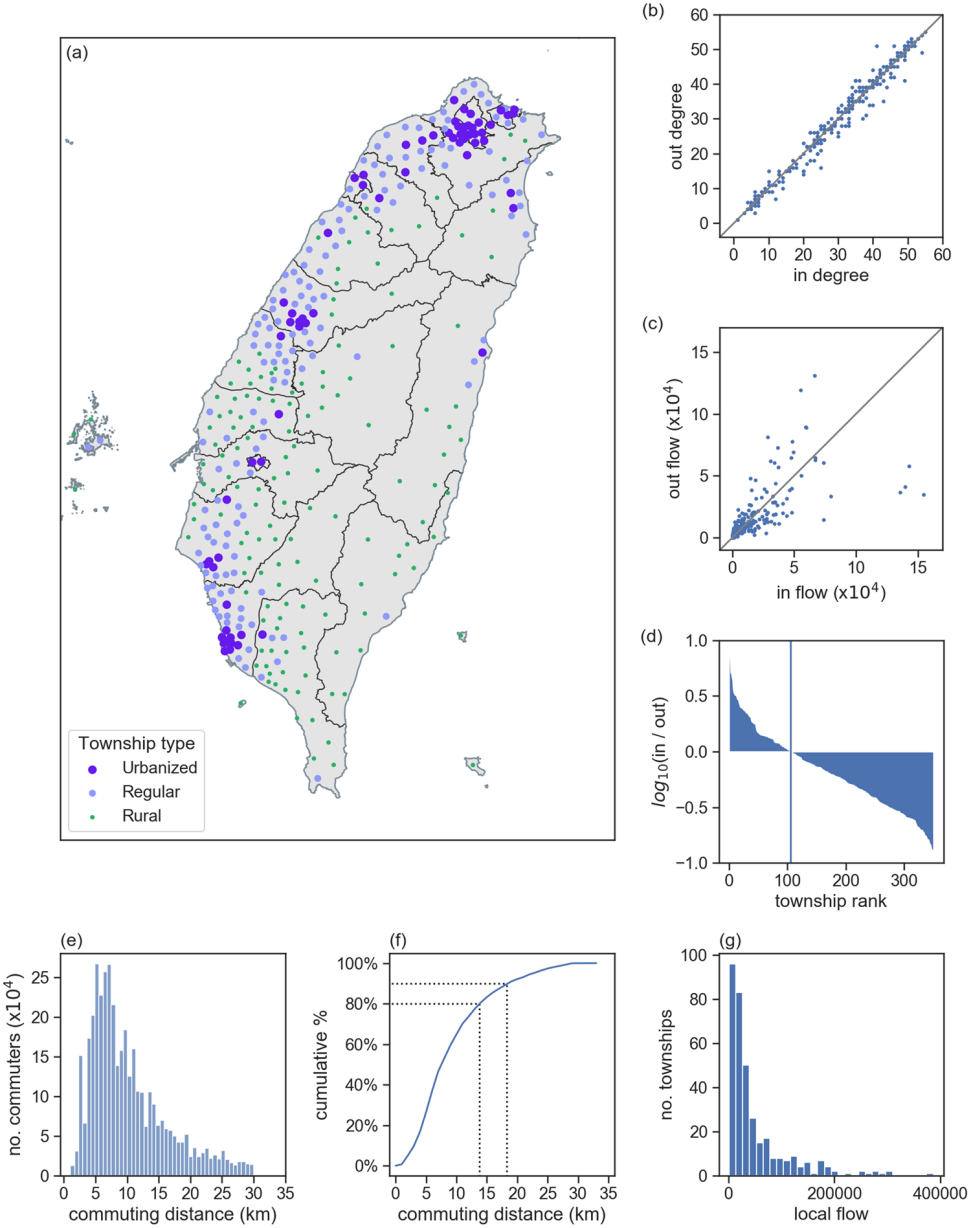
Commuter flow data for Taiwan’s main island. (**a**) Circles indicate three township density levels: urbanized (purple), regular (blue) and rural (small green dots). (**b**) Numbers indicate in-degree and out-degree values for each township. (**c**) Incoming and outgoing commuter numbers for individual townships (i.e., weighted in- and out-degrees). (**d**) Log ratios (*log*_10_) for incoming to outgoing commuter numbers, with blue vertical line demarcating zero log ratios. Left side townships have more incoming than outgoing commuters (greater pulling strength); right side townships have more outgoing than incoming commuters (greater pushing strength). (**e**) Commuting distance distribution data for 3,906,663 inter-township commuters, showing that the large majority travel between 5 and 15 km one-way on a daily basis. (**f**) Cumulative percentages of commuters in terms of travel distance, showing that 80% travel 14 km or less and 90% 18 km or less. (**g**) Frequency distribution data for commuters who work and live in the same township.

The incoming and outgoing statistical-plus-spatial urbanization pattern data used in this study are shown in Fig. 2, with Fig. 2a. specifically showing degree of township urbanization. Liu *et al*.^42^ describe seven township levels in Taiwan. For visualization purposes we collapsed four to create three: urbanized (highly and moderately urbanized cities), regular (emerging and regular towns), and rural (agricultural and remote areas). Concentrated urban areas are Taipei (north Taiwan), Taichung-Changhua (middle west coast), and Tainan-Kaohsiung (far southwest). Figure 2b shows origin and destination township numbers, and Fig. 2c shows the numbers of commuters entering and leaving townships for work or school. Note that the Fig. 2c data have a skewed distribution with low commuter numbers (50,000 maximum). Figure 2d shows in-out ratio logarithm data in descending order. Log ratios >0 (106 townships) indicate a large number of incoming and small number of outgoing commuters, and log ratios <0 (245 townships) indicate larger numbers of individuals leaving than entering townships. As shown, most townships have negative log ratio values, meaning they “push” commuters to larger urban areas; townships with positive log ratio values are said to “pull” commuters. The longest commuting distance for the 3,906,663 individuals examined for this study was 30 km (Fig. 2e), with 90% travelling less than 18 km and 80% less than 14 km (Fig. 2f). Compared to many other countries, commuting distances in Taiwan are much shorter. Frequency distribution data for commuters living and working in the same township are shown in Fig. 2g. For all townships considered in this study, an average of 84% (SD 7%) of each population resided and worked in the same township.

### Disease data

The two infectious diseases used for making comparisons with actual case distributions were the H1N1 influenza virus and the type 71 enterovirus (EV). Data were collected from the Taiwan National Infectious Disease Statistics System maintained by the Taiwan Center for Disease Control, a database with records for various types of notifiable diseases going back to 1999. We gathered data for 1,129 H1N1 cases reported in the 353 townships in 2009. Epidemic risk was defined as the number of flu cases in each township. For EV we used data for five years in which at least 100 severe cases were reported—2000 (291 cases), 2001 (393), 2002 (162), 2005 (142) and 2008 (373). Epidemic risk was defined as the average number of EV cases per year per township.

**Figure 3:**
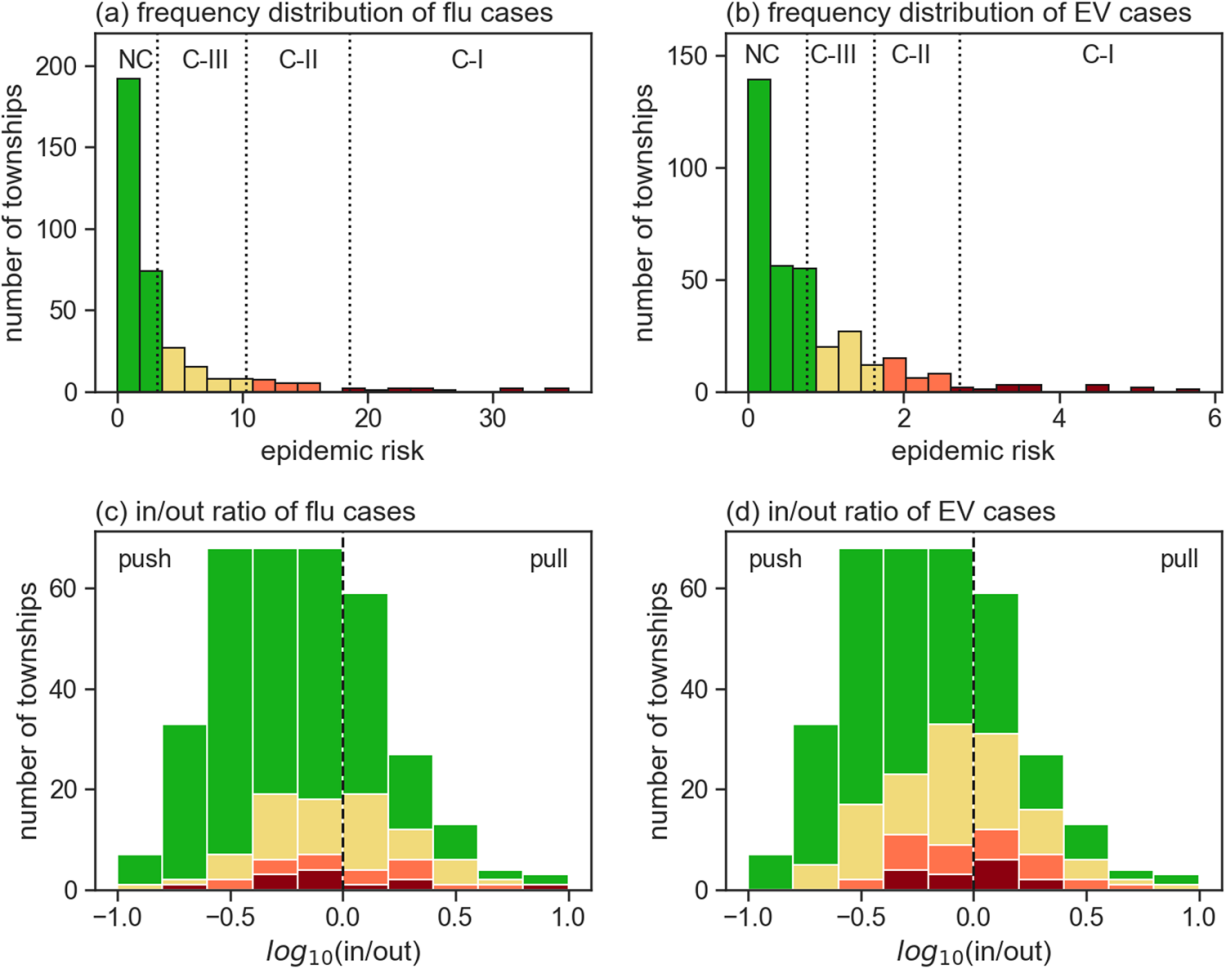
Frequency distributions for (**a**) reported H1N1 flu cases and (**b**) average numbers of Type-71 enterovirus cases for 2000, 2001, 2002, 2005 and 2008 combined. Distributions were categorized as non-core (NC, green), core-III (yellow), core-II (orange), and core-I (red) based on head/tail breaks. Second row shows township frequencies according to in-out logarithm ratios.

Figure 3a,b show frequency distributions for the two diseases, and Fig. 3c,d show corresponding epidemic levels according to flow patterns (in-out ratio logs). The figures show skewed distributions for both diseases (from left-to-right: non-core, core-III, core-II and core-I). The skewed patterns generally suggest lower numbers of flu and EV cases in most townships, and only a small number of townships with high epidemic risk. Head/tail breaks^43^ were used to differentiate among epidemic risk levels across townships (three breaks per disease). This method is useful for separating data into various groups when frequency distributions are exponential or skewed. Specifically, head/tail breaks were initially used to separate frequency distributions according to the means of whole data sets, with iterated means expressed on the right side of each division. The second row figures show the distributions of four epidemic levels. According to these distributions, the core-I to core-III townships were a combination of pull and push types—in other words, they were located on both sides of the dotted line demarcating a zero log ratio.

**Figure 4:**
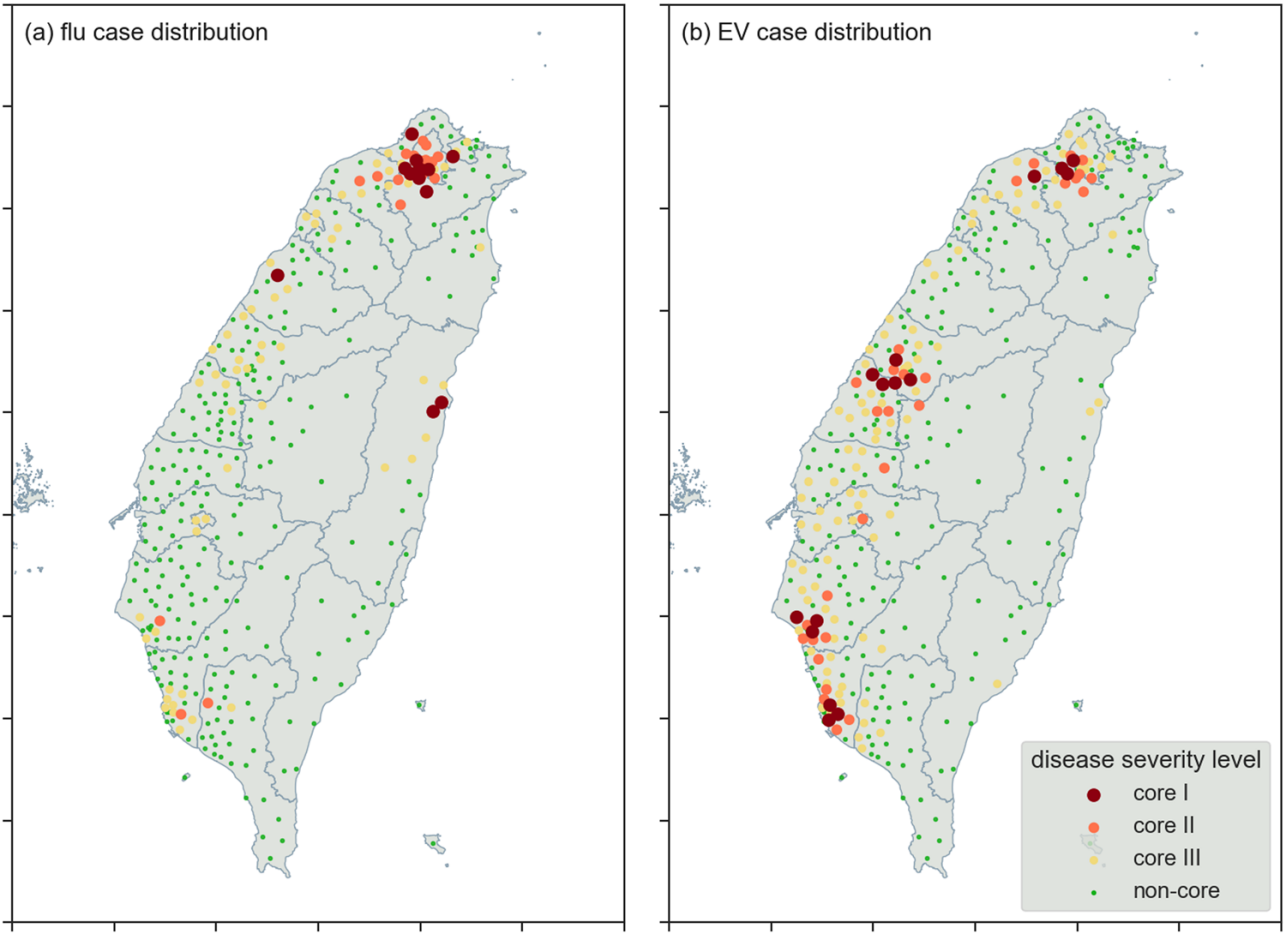
Maps showing spatial distributions for (**a**) H1N1 flu, which spreads via droplets and (**b**) Type-71 enterovirus, which spreads via physical contact. Township disease case severity levels are categorized as core-I (red), core-II (orange), core-III (yellow) and non-core (green).

Spatial distributions for the four epidemic levels for each disease are presented in Fig. 4. As shown, flu cases were more concentrated in sections of the Taipei metropolitan area, while EV cases were concentrated in Taiwan’s four largest cities: Taipei, Taichung, Tainan and Kaohsiung.

### The EpiRank algorithm

**Figure 5:**
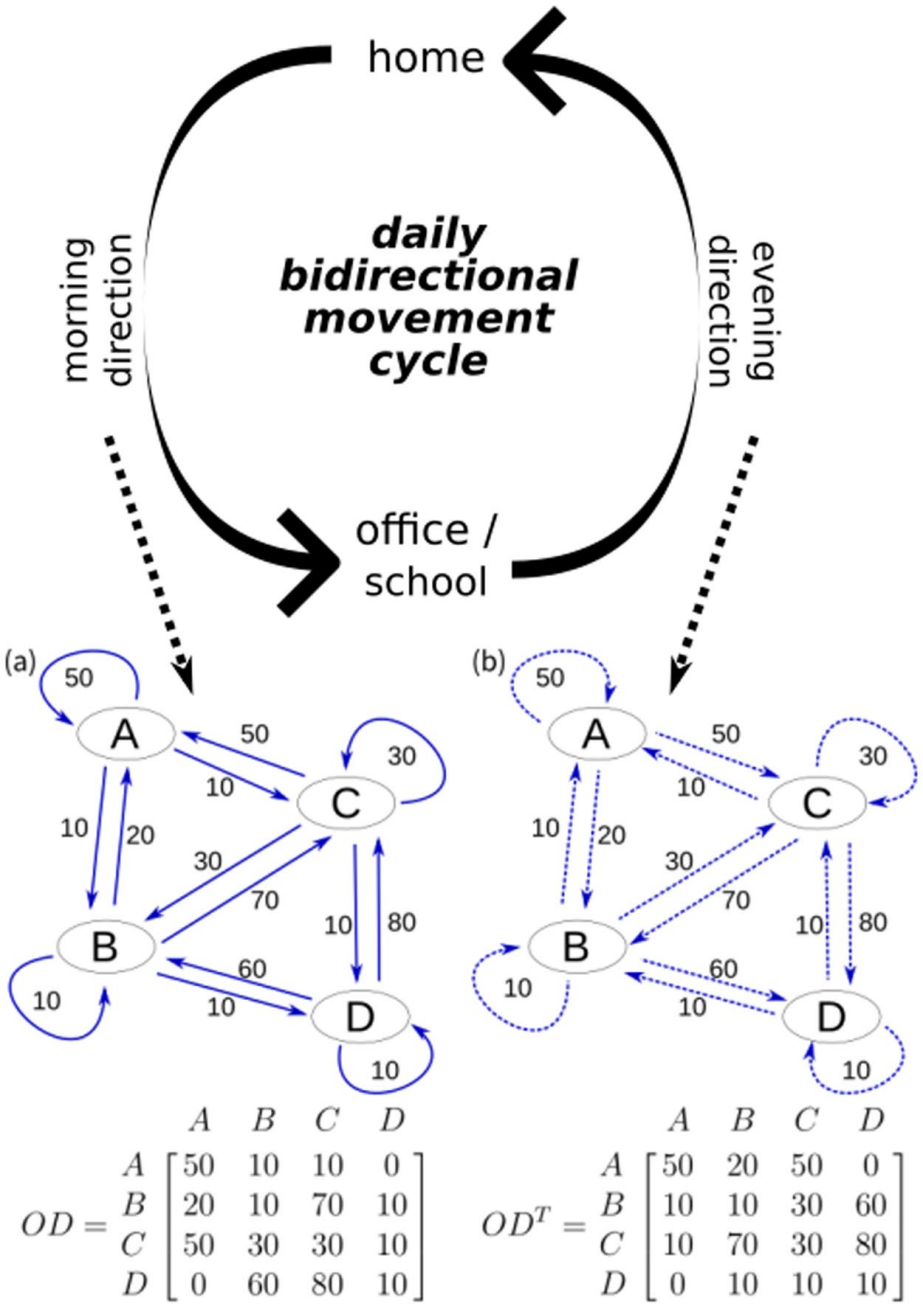
Daily bidirectional movement cycle. Shown are forward (A to B) and backward (B to A) movement flow networks and corresponding OD-matrix.

The Markov chain model and PageRank were used to create an algorithm for considering both forward and backward movement in commuting networks to calculate epidemic risk in individual townships due to a transitive effect. An example of commuting flow between four regions is shown in Fig. 5. Using region A as an example, the potential risk of disease spread is associated with three infectious paths: (a) the 20 individuals from B and 50 from C who travel to A for work (incoming commuters), (b) the 20 who leave A and travel to B or C and return to A in the evening (returning commuters), and (c) the 50 who work or attend school and live in the same region, thus increasing the potential for intra-region infections (local commuters).

To describe how EpiRank functions, assume a commuting network *G*(*v*,*e*) with |*v*| = *n* and |*e*| = *m*, indicating *n* townships and *m* commuting relationships between townships. For any township i, an EpiRank value *ER*(*i*) emerges from a large *t* number of iterations involving the following equations:

1 $$ER\,{(i)}^{t+1}=(1-d)\times exFac\,(i)+d\times (FW{(i)}^{t}+BW{(i)}^{t})$$

2 $$FW{(i)}^{t}=daytime\times {\sum }_{j}(\frac{O{D}_{j,i}}{{\sum }_{k}O{D}_{j,k}}\times ER{(j)}^{t})$$

3 $$BW{(i)}^{t}=(1-daytime)\times \sum _{j}(\frac{O{D}_{i,j}}{{\sum }_{k}O{D}_{k,j}}\times ER{(j)}^{t})$$

where *ER*(*i*)^*t*+1^ and *ER*(*i*)^*t*^ are the *ER* values for region *i* in iterations *t* + 1 and *t*, respectively; *d* is the effect of the network topological structure on epidemic risk within a range of 0 and 1 (0.95 default value in this study); (1 − *d*) captures the disease spreading effect associated with local environmental factors rather than network topological structure; *exFac*(*i*) is an unspecified external effect influencing epidemic risk (1*/n* default value in this study); *FW*(*i*)^*t*^ and *BW*(*i*)^*t*^ are the effects of forward and backward movement, respectively; and *daytime* is a 0−1 parameter controlling differences between *FW*(*i*) and *BW*(*i*), with a higher *daytime* value indicating more forward than backward movement and vice versa. The OD matrix records connectivity from residential (rows) to work townships (columns). *OD*_*j*,*i*_ indicates flow from region-*j* (origin) to region-*i*, and ∑_*k*_ *OD*_*j*,*k*_ is the total number of commuters originating from region-*j*.

The above equations illustrate the EpiRank transitive effect and calculation processes for each township. An EpiRank value for the entire network is calculated using a matrix multiplication approach involving three equations:

4 $$E{R}^{t+1}=(1-d)\times exFac+d\times (F{W}^{t}+B{W}^{t})$$

5 $$F{W}^{t}=daytime\times ({W}^{T}\times E{R}^{t})$$

6 $$B{W}^{t}=(1-daytime)\times (W\times E{R}^{t})$$

where *ER*^*t*+1^ and *ER*^*t*^ are *ER* distribution values for iterations *t* + 1 and *t*, respectively, in a *n* × 1 matrix; *ER*^0^ is the initial status, set to 1*/n*; *exFac* denotes external factors that may affect *ER* value (factors taking the form of a *n* × 1 matrix with a default value of 1*/n* for each cell or standardized column, indicating differences in external factors between townships); *FW*^*t*^ and *BW*^*t*^ are the effects of forward and backward movement; *W* is a standardizing matrix for each column in the OD-matrix; and *W*^*T*^ is a standardizing matrix following transposition.

Since the calculation concept follows the Markov chain mechanism and PageRank calculation procedures, the summation of all *ER* values (representing random commuter distributions within each analyzed network in each iteration) equals 1.

The following matrix shows calculations based on the Fig. 5 example. *ER* values during iteration *t* are shown as *a*, *b*, *c* and *d*. The EpiRank value for the four areas is the summation of *BW*^*t*^ and *FW*^*t*^. *FW*^*t*+1^ is calculated as

7 $$\begin{array}{rcl}F{W}^{t+1} & = & daytime\times [\begin{array}{cccc}5/7 & 2/11 & 5/12 & 0\\ 1/7 & 1/11 & 3/12 & 6/15\\ 1/7 & 7/11 & 3/12 & 8/15\\ 0 & 1/11 & 1/12 & 1/15\end{array}]\times [\begin{array}{c}a\\ b\\ c\\ d\end{array}]\\  & = & daytime\times [\begin{array}{c}5/7(a)+2/11(b)+5/12(c)+0(d)\\ 1/7(a)+1/11(b)+3/12(c)+6/15(d)\\ 1/7(a)+7/11(b)+3/12(c)+8/15(d)\\ 0(a)+1/11(b)+1/12(c)+1/15(d)\end{array}]\end{array}$$

A similar procedure with a transposed standardized column matrix was used to calculate *BW*^*t*+1^. *BW*^*t*+1^ and *FW*^*t*+1^ are both *n* × 1 matrixes.

8 $$\begin{array}{rcl}B{W}^{t+1} & = & (1-daytime)\times [\begin{array}{cccc}5/12 & 1/11 & 1/19 & 0\\ 2/12 & 1/11 & 7/19 & 1/3\\ 5/12 & 3/11 & 3/19 & 1/3\\ 0 & 6/11 & 8/19 & 1/3\end{array}]\times [\begin{array}{c}a\\ b\\ c\\ d\end{array}]\\  & = & (1-daytime)\times [\begin{array}{c}5/12(a)+1/11(b)+1/19(c)+0(d)\\ 2/12(a)+1/11(b)+7/19(c)+1/3(d)\\ 5/12(a)+3/11(b)+3/19(c)+1/3(d)\\ 0(a)+6/11(b)+8/19(c)+1/3(d)\end{array}]\end{array}$$

In these calculations, *FW*^*t*+1^ uses an *OD*^*T*^ standardized column matrix (*W*^*T*^) and *BW*^*t*+1^ uses an OD standardized column matrix (*W*), since the matrix multiplication process in the Markov chain model puts the flow matrix (*n* × *n*) at the front, followed by the previous *ER* value from iteration *t* (an *n* × 1 matrix). To ensure that the matrix multiplication produces the same results as the above-described EpiRank Eq. 1 for a single township, the matrix columns should represent origin townships and matrix rows destination townships (a transposed OD-matrix). For directed links, bidirectional flows consist of the same groups of people. Differences in the forward and backward effects on links involve direction movement and a denominator. For the forward effect the denominator is the total number of individuals leaving from the location of origin; for the backward effect the denominator is the total number of individuals leaving from the original destination.

Using the example shown in Fig. 5, for the forward movement assume 50 commuters moving from C to A. The sum of the C column in the transposed OD matrix (*OD*^*T*^) is 120 (meaning 120 commuters leave C), hence the influence from C to A is 50*/*120 the current risk status in C. For the backward movement, the 10 commuters returning from C to A are the same 10 who moved from A to C in the morning; accordingly, the sum of the C column in the pre-transposed OD matrix is 190, meaning that 190 individuals travelled to C in the morning and left C in the evening, making the C-to-A influence 10*/*190 the current C risk status. In summary, the total influences on A resulting from forward movement is *daytime* × (5*/*7(*a*) + 2*/*11(*b*) + 5*/*12(*c*) + 0(*d*)), and the total influence on A resulting from backward movement is (1−*daytime*) × (5*/*12(*a*) + 1*/*11(*b*) + 1*/*19(*c*) + 0(*d*)). These calculations can be performed using the above-described matrix multiplication process.

Continuing with the above example, EpiRank value calculations for all iterations are shown below, with *daytime* set to 0.5, *d* to 0.95, and *exFac* for each region set to (1*/n*). Results from left-to-right are for *t* = 0, 1, 2, 3, 4, 5, …, 47. If precision is set to 3 decimal places, the Markov chain model enters a steady state at *t* = 5; if precision is set to 8 decimal places, it enters a steady state at *t* = 47. In this example, EpiRank values have the highest concentration at c, followed by a, b and d.

9 $$[\begin{array}{c}a\\ b\\ c\\ d\end{array}]=[\begin{array}{c}0.250\\ 0.250\\ 0.250\\ 0.250\end{array}]\to [\begin{array}{c}0.235\\ 0.231\\ 0.338\\ 0.195\end{array}]\to [\begin{array}{c}0.244\\ 0.234\\ 0.321\\ 0.201\end{array}]\to [\begin{array}{c}0.246\\ 0.233\\ 0.323\\ 0.198\end{array}]\to [\begin{array}{c}0.247\\ 0.233\\ 0.323\\ 0.198\end{array}]\to [\begin{array}{c}0.247\\ 0.233\\ 0.323\\ 0.198\end{array}]\to \cdots \to [\begin{array}{c}0.247\\ 0.233\\ 0.322\\ 0.198\end{array}]$$

In summary, we modified the PageRank algorithm to include bidirectional movement to capture pathogen infection risk within a topological network structure involving both forward and backward movement. We separated EpiRank values into two parts (*FW* and *BW*) for Markov chain model calculations, and integrated them using a daytime parameter to control their weighted effects. In addition to movement between regions, we also included movement within each region, as indicated by the diagonal lines in both of the Fig. 5 OD matrixes. The added *exFac* can be used to consider the collective effects of other factors that are not related to network topological structure. *exFac* is integrated into calculations via a damping factor (the (1−*d*) in Eqs 1 and 4), with a default value of 1*/n* for all regions, indicating an assumption of equal distribution across all townships.

## Results

This study consisted of three parts. In the first we compared risk distribution according to three daytime settings (0, 0.5 and 1) respectively representing the transitive effects of backward-only, bidirectional, and forward-only movement. In the second part, EpiRank results were compared to actual data for two infectious diseases. In the third part we compared bidirectional EpiRank results with those produced by three other network indexes, and tested the sensitivity of daytime and damping factor parameters.

### Transitive effects of forward, backward and bidirectional movement

**Figure 6:**
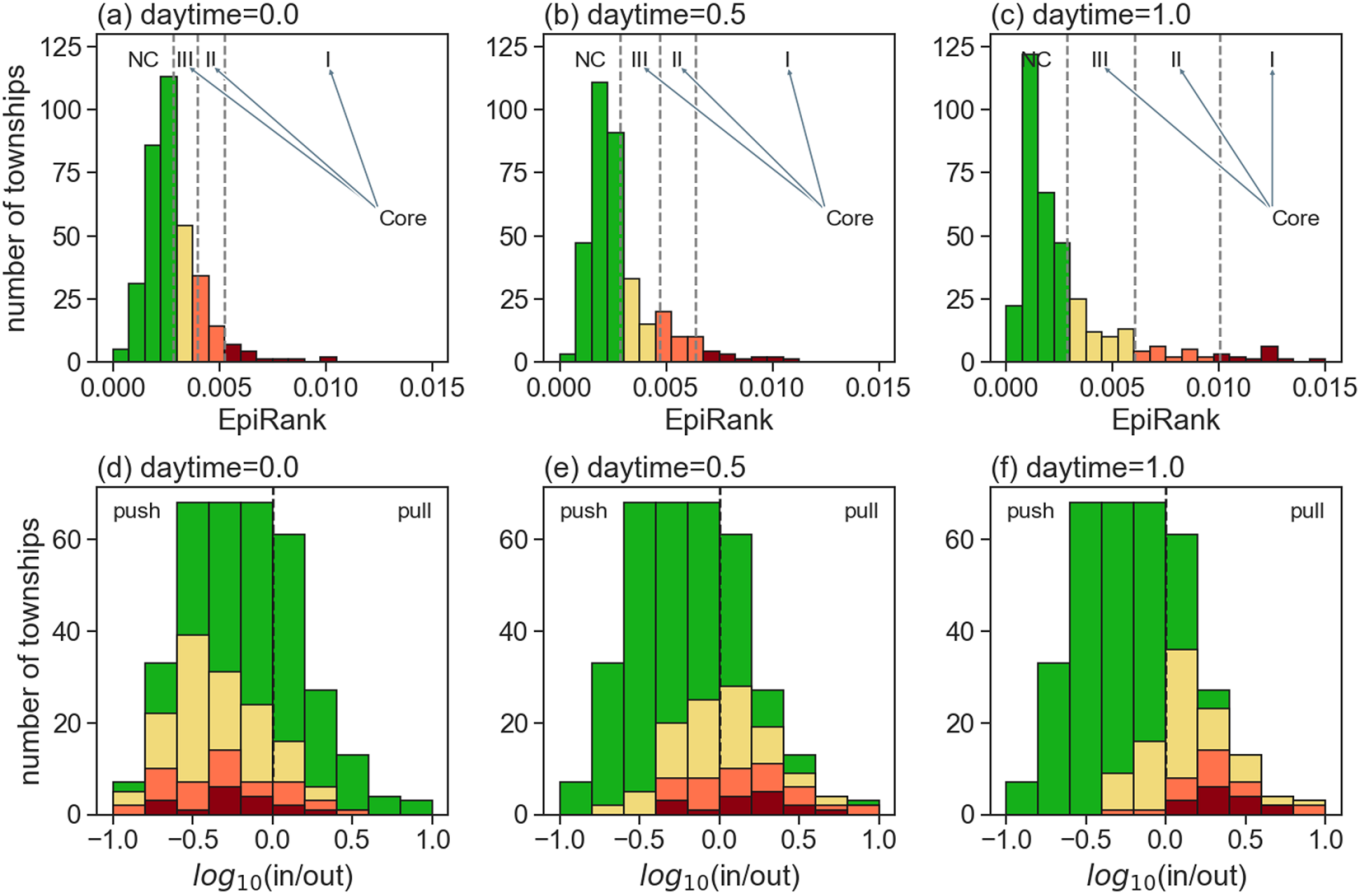
Frequency distributions according to EpiRank values (first row) and log in-out ratios (second row). (**a**) Daytime = 0, meaning that only backward movement is considered; **(b**) daytime = 0.5, meaning that forward and backward movement are both considered (equal effects for each direction); (**c**) daytime = 1.0, meaning that only forward movement is considered; (**d**) distributions for four levels according to log in-out ratios for daytime = 0, with core townships primarily located in areas with low ratios (greater pushing than pulling power); (**e**) core townships primarily located in areas with comparable numbers of inward and outward-moving commuters; (**f**) core townships with greater pulling than pushing properties.

As stated in an earlier section, a 0 *daytime* setting indicates that only backward movement is being considered (with the *FW* effect completely removed from calculations) and a 1 *daytime* setting indicates that the *BW* effect is completely ignored in favor of forward movement. A 0.5 setting indicates equal weights for forward and backward movement in EpiRank calculations. Results from those calculations are shown in Fig. 6 (frequency distributions) and 7 (spatial distributions). Since the Fig. 6 data are skewed, we used head/tail breaks to group townships as non-core, core-III, core-II and core-I, similar to the procedure for grouping the two diseases. The most skewed pattern among the three results is found in Fig. 6c, indicating more non-core townships and fewer core-I townships.

**Figure 7:**
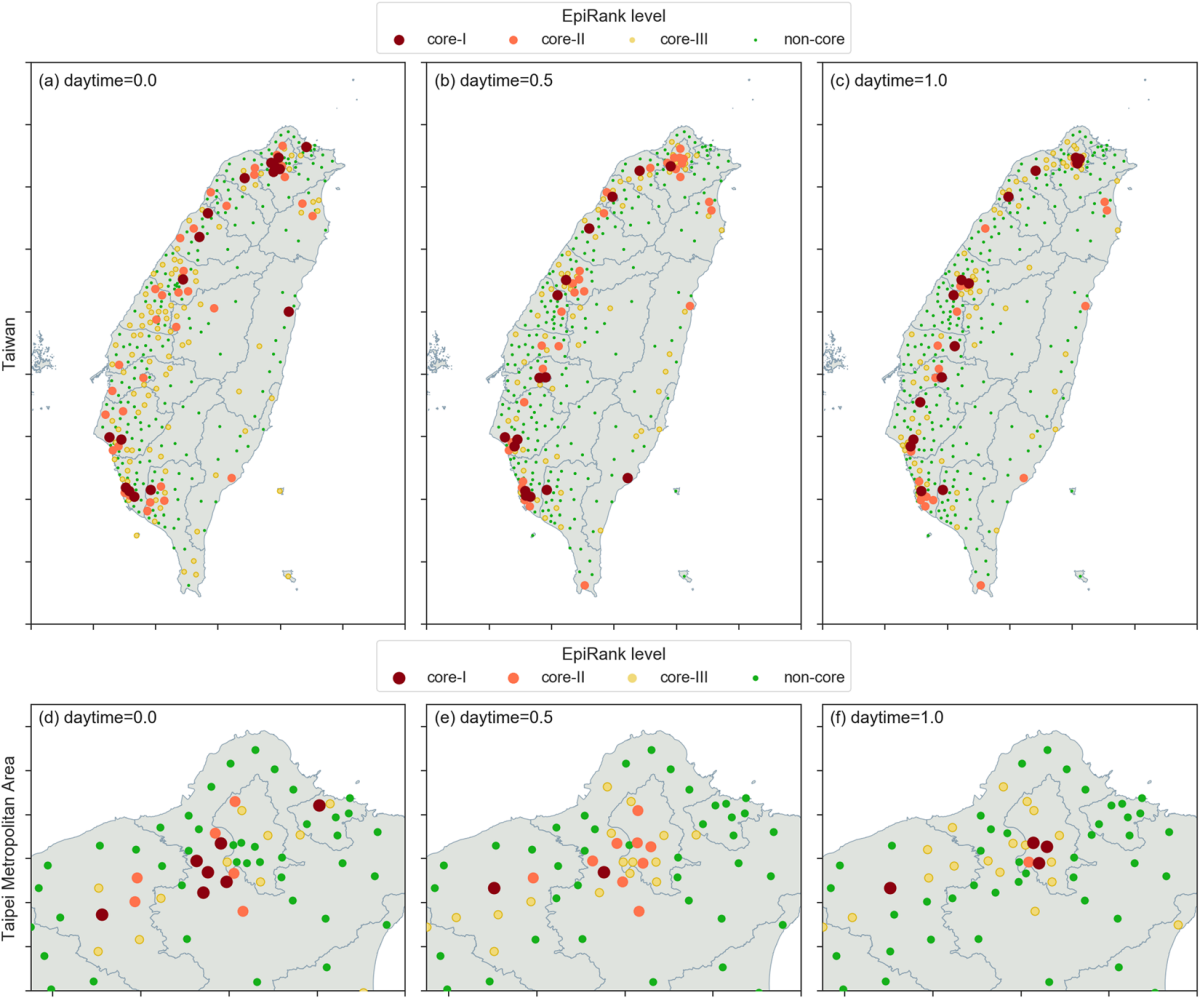
EpiRank spatial distribution values for all of Taiwan (first row) and the Taipei Metropolitan Area only (second row). Figures a and d show results for daytime = 0.0, figures (**b**,**e**) daytime = 0.5, and figures (**c**,**f**) daytime = 1.0.

In Fig. 7, core-I townships are shown as the largest circles (red), core-II as the second largest (orange), and core-III the third largest (yellow); green dots indicate non-core townships. The spatial distribution for all of Taiwan is presented in the first row (a-c), and for the Taipei metropolitan area (including Taipei, New Taipei, Keelung and Taoyuan) in the second row. As shown, major forward commuting network flows are from residential to central business districts (CBDs), and major backward flows are from CBDs to residential districts in neighboring or satellite regions. When only backward movement is considered in the Taipei metropolitan area (Fig. 7d), high-ER value (core-I) townships are mostly concentrated in the southwest section of New Taipei; in contrast, most Taipei townships are core-III. This pattern extends to Taoyuan (southwest of the core Taipei area), which has higher numbers of core-II and core-III townships. When only forward movement is considered (Fig. 7f), core-I townships are mostly concentrated in the southwest section of Taipei and extend further southwest to both New Taipei and Taoyuan, where there are more core-II and core-III townships. When bidirectional movement is considered (Fig. 7e), Taipei townships are predominantly core-I and core-II, and once again higher numbers of core-II and core-III townships are found in New Taipei and Taoyuan.

Similar observations were made for the Taichung-Changhua metropolitan area in west-central Taiwan and the Tainan-Kaohsiung cluster in the far south. Specifically, when only forward movement is considered, core townships (especially core-I townships) are concentrated in central urban locations (Fig. 7c), and when only backward movement is considered, core areas are scattered throughout satellite regions (Fig. 7a). When bidirectional movement is considered, first-level core townships are primarily located in central urban locations, while a larger number of second- and third-level core townships are found in neighboring regions.

The forward-only and backward-only cases represent the one-way transmission of pathogens toward or away from CBDs. Greater disease spread during morning hours indicates higher rates of transmission in or during movement toward CBDs; higher transmission rates during evening hours occur as commuters move toward home/satellite regions. These observations are also clear in Fig. 6d,f, with concentrated core townships exhibiting pushing properties when backward movement alone is considered, and pulling properties when only forward movement is considered. Townships with pushing properties are more likely to be in residential areas, and townships with pulling properties are more likely to be in commercial districts. During disease outbreaks, disease spread may influence both townships where individuals live and where they work. For this reason, the distribution of results based on a 0.5 *daytime* parameter exhibited similar patterns for the two diseases.

### Comparison of bidirectional EpiRank and disease data

**Figure 8:**
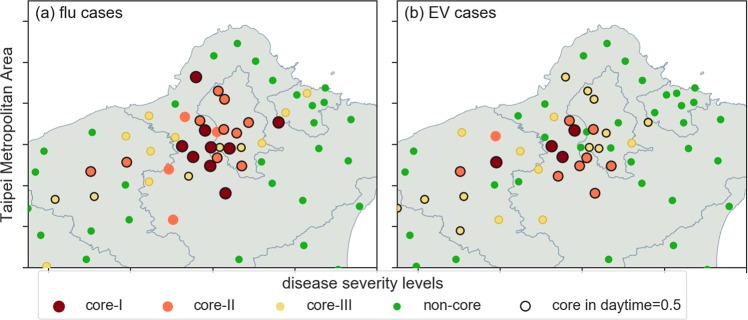
Comparison of core townships identified by EpiRank (black circles) and core/non-core township distributions for (**a**) H1N1 flu cases and (**b**) Type-71 enterovirus cases in the Taipei Metropolitan Area. Calculations based on daytime = 0.5.

The actual flu and EV distribution data shown in Fig. 8 were compared with predicted conditions. Black circles indicating townships identified by EpiRank as core at all three levels plus actual data for both diseases were added to Fig. 8a,b. As shown, core flu case townships are clustered in the southwest sections of Taipei and New Taipei, with all Taipei townships identified as core. In contrast, the EV case distribution shown in Fig. 8b has an extended pattern in which core-I and core-II townships appear in the southwest section of New Taipei only; in contrast, Taipei was limited to core-II and core-III townships. A possible explanation is that most EV cases resulted from closer and longer interactions between infected and susceptible individuals.

**Figure 9:**
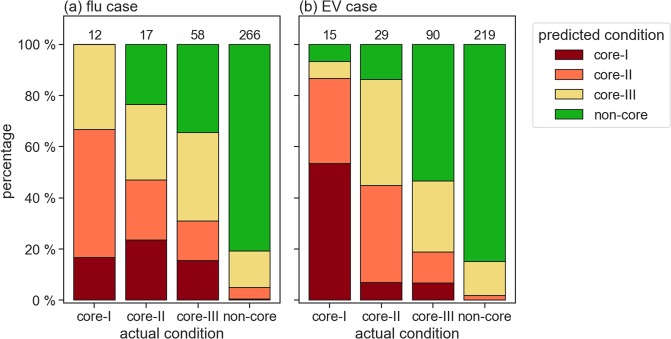
Predicted cases for core and non-core townships expressed as percentages of actual cases for (**a**) H1N1 flu and (**b**) the Type-71 enterovirus. Numbers at top of each bar indicate total numbers of actual cases.

Figure 9 presents a comparison of actual and EpiRank-predicted conditions. Green bars indicate predicted non-core percentages of the four actual conditions. According to the first bar in Fig. 9a, of the 12 townships in the core-I flu case group, none were identified by EpiRank as non-core and 8 were identified by EpiRank as either core-I or core-II (66.7%). The percentage of EpiRank-identified non-core townships increased for actual core-II townships and even more for actual core-III townships. The same data also indicate that recall (i.e., true positives divided by total numbers of core townships) decreased for township groups with lower epidemic risk values (100% for core-I, 76.5% for core-II, and 67.9% for core-III). Combined, EpiRank correctly identified 63 of 87 actual core-I, core-II and core-III flu case townships (72.4% recall). Among the 266 actual non-core townships, 215 were identified by EpiRank as non-core—an 80% level of specificity (true negatives divided by total numbers of non-core townships). Similar patterns were found for EV cases: 93.3%, 86.2% and 70.0% recall values for actual core-I, core-II and core-III townships, respectively. The combined results show that EpiRank correctly identified 81 of 134 (60.4%) actual core-I, core-II and core-III EV case townships—that is, they indicate that EpiRank exhibited low likelihoods of under- and overestimation.

### Comparisons with previous methods

Cumulative results for transitive effects in a commuting network can be determined using network measures such as PageRank and HITS (including both Hub and Authority, hereafter referred to as HITS-Hub and HITS-Authority). Head/tail breaks were used to organize results from the three non-EpiRank indexes according to the four core/non-core township categories (Table 1). In all, PageRank identified 107 townships as core-I, core-II or core-III, and 246 as non-core. Hit-Hub identified 26 core and 327 non-core townships, and HITS-Authority identified 29 core and 324 non-core townships.

**Table 1:** Numbers of core/non-core townships identified by EpiRank, PageRank, HITS-Hub and HITS-Authority.

	EpiRank	PageRank	HITS-Hub	HITS-Authority
core-I	16	15	1	1
core-II	31	20	2	3
core-III	67	72	23	25
non-core	239	246	327	324

We used Pearson and Spearman correlation coefficients and two binary classification tests (recall and precision) to compare distribution results generated by the three network measures and EpiRank for both diseases. As shown in Table 2, correlations for the EpiRank and actual disease data were higher than those for the other three network metrics, and EpiRank recall values were highest among the four. A possible explanation for EpiRank’s lower precision compared to HITS-Hub and HITS-Authority is the significantly smaller number of core townships in the latter two. Precision is defined as the proportion of true positives to the total number of detected cores (i.e., true predicted condition), therefore the smaller numbers of core townships may have produced higher precision values for the two HITS indexes.

**Table 2:** Correlations between actual cases and results from four network indexes, including two correlation coefficients (Pearson and Spearman) and two binary classification tests (recall and precision).

Disease	Index	EpiRank	PageRank	HITS-Hub	HITS-Authority
Flu	Pearson’s R	0.513	0.355	0.387	0.397
	Spearman’s Rho	0.531	0.445	0.4	0.403
	Recall	0.724	0.609	0.299	0.333
	Precision	0.553	0.495	1	1
EV	Pearson’s R	0.706	0.536	0.217	0.219
	Spearman’s Rho	0.599	0.53	0.194	0.199
	Recall	0.605	0.515	0.164	0.172
	Precision	0.711	0.645	0.846	0.793

**Figure 10:**
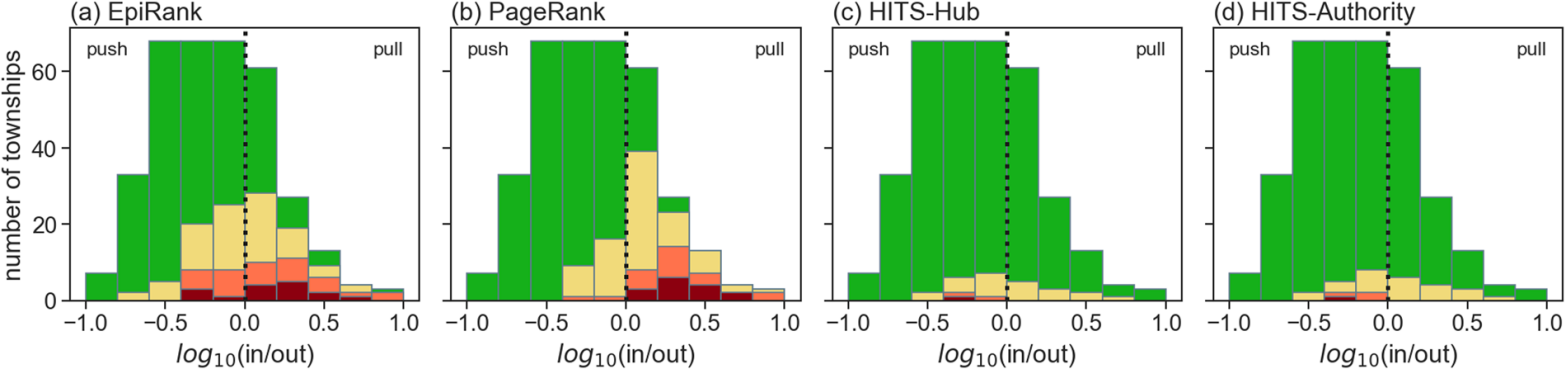
Distribution results from (**a**) EpiRank, (**b**) PageRank, (**c**) HITS-Hub and (**d**) HITS-Authority expressed as log in/out ratios. Non-core and core areas are shown in green and red, respectively.

Core and non-core township distributions according to log in-out ratios are presented in Fig. 10. As shown, PageRank results concentrated core townships on the right side of the graph, indicating that most had pull properties (Fig. 10b). In contrast, HITS-Hub (Fig. 10c) and HITS-Authority (Fig. 10d) produced higher numbers of core townships on the left side, indicating more push properties. Our EpiRank distribution results were close to actual data for both diseases (Fig. 3). EpiRank outperformed the other three network indexes for identifying push townships, as well as for identifying non-core townships.

### Daytime and damping factor parameter sensitivity

**Figure 11:**
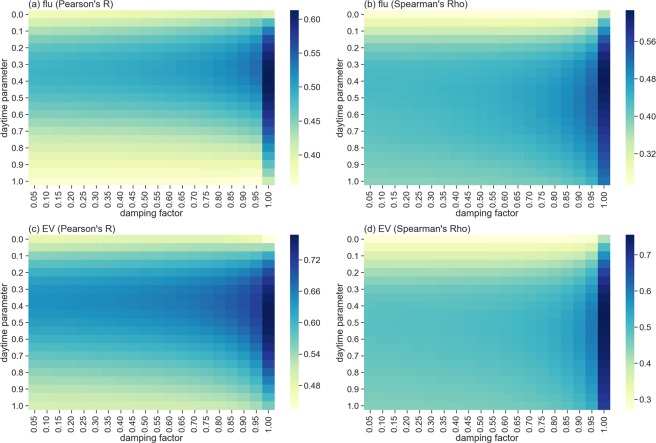
Validation sensitivity data according to daytime (forward and backward movement) and damping factor values (i.e., effects of network topology compared to external factors). (**a**) Pearson’s R for flu cases, (**b**) Spearman’s rho for flu cases, (**c**) Pearson’s R for EV cases, (**d**) Spearman’s rho for EV cases.

The two primary EpiRank parameters are *daytime* (for controlling weights between forward and backward effects) and *damping factor* (for controlling weights between network transitive and external factor effects). The *daytime* parameter value indicates the strength of the forward movement effect, and the 1−*daytime* value the strength of the backward movement effect. Results from sensitivity tests for the two parameters are shown in Fig. 11. The *daytime* value ranged between 0 and 1 in 0.05 increments (21 individual values). The EpiRank damping parameter *d* refers to the network topological structure weight, with (1−*d*) indicating the influence of one or more external factors. A damping factor of 0 indicates no effect of network topological structure on distribution, and a damping factor of 1 indicates no effect from one or more external factors on disease diffusion. The external factor distribution in this study was equal for all townships, meaning that EpiRank values for all nodes were equal when the damping factor was set to 0. For our sensitivity analyses we set the damping factor from 0.05 to 1.00 in 0.05 increments (20 individual values).

EpiRank results for the two diseases were compared using Pearson and Spearman correlation coefficients. As shown in Fig. 11a–d, correlations initially increased in step with *daytime* value increments, then started to fall between values of 0.4 and 0.5. Flu case correlations peaked at *daytime* values of 0.3 (Pearson’s R) and 0.5 (Spearman’s rho), and EV case correlations peaked at *daytime* values of 0.4 (Pearson’s R) and 0.5 (Spearman’s rho). Combined, these results indicate that EpiRank captured the structure of bidirectional movement. According to the Y-axis data in the four Fig. 11 graphs, all correlation peak data for *daytime* values were slightly below 0.5, indicating that the backward movement effect was more important than the forward movement effect—in other words, disease spread was more likely to occur during backward than forward commuting movement. EpiRank successfully captured this effect. Our finding that lower *daytime* values were better at capturing disease distribution may be due to interaction diversity and intensity. Individuals who interact with large numbers of colleagues, clients, classmates, or others during work hours have many low-intensity interactions, while individuals who stay at home with small numbers of family members or roommates have low diversity but high intensity interaction values, resulting in very different disease transmission effectiveness rates. When *daytime* values are lower than 0.5, the strength of the backward effect exceeds that of the forward effect, indicating greater importance for interaction intensity compared to diversity.

The correlation coefficients for the X-axis dimension indicate slow but steadily increasing values in step with increased damping, with the highest correlations for both flu and EV observed at a factor value of 1.0. In sum, correlation changes associated with the damping factor were much smaller than those associated with the *daytime* factor, possibly because differences in external factors between townships were not considered, thereby increasing the effect of network topological structure on disease spreading.

## Discussion and Conclusion

Previous researchers have used networks to capture topological structures underlying disease diffusion^44–47^ or to model different disease control strategies and consider their potential outcomes^30,31^. Network structures have also been used to analyze inter-city movement and transportation networks^14,40^, multi-layer interactions between cities^48,49^, surface street congestion problems^15,50,51^, airline flight patterns^52–54^, and maritime movement^53,55,56^. Our proposed algorithm offers a novel perspective for these and other networks.

Network topological structure diffusion studies have generally emphasized the forward movement of directed links^30,57^. Some researchers have investigated car movements in street networks to understand congestion patterns and to identify locations where cars and pedestrians gather as the transitive results of those movements^14,15^. Commuting networks capture morning (forward) movement from where people live to where they work or attend school, and evening (backward) movement in the opposite direction. Commuting network studies have generally neglected this combination and its implications. Our motivation to create the EpiRank algorithm was to capture the forward and backward movement effects missed by previous methods.

In epidemiological studies, compartment models such as SIR or SEIR are frequently employed to analyze the temporal dimension of disease outbreaks. Although compartment models are useful for analyzing dynamic changes in disease events over time, the addition of spatial considerations generally makes them excessively complex. Epidemic models such as EpiSimS^45,58^, GLEAMviz^59,60^, and EpiFast^61^ were created to add spatial variation and human movement into analyses by inserting other factors and processes into disease diffusion models. The strength of these models is their ability to produce detailed results with high degrees of accuracy, but their weakness is the high costs of acquiring, processing and working with input data. For our proposed EpiRank algorithm, input data primarily consist of the OD flow matrixes of commuting networks, and analyses consist of low-cost matrix multiplication series. As an example, for a scenario consisting of 353 nodes (townships) and 11,220 links (commuting connections), only 588 EpiRank iterations were required to achieve a stable outcome. While EpiRank cannot capture the complex and detailed epidemiological structures of disease diffusion, it is more than adequate for capturing geographic diffusion outcome patterns in broader contexts. Accordingly, EpiRank may have high utility for public health authorities working on resource distribution problems during disease outbreaks.

Although we focused on two infectious diseases that are spread via droplets and physical contact, the spread of mosquito-borne diseases such as the dengue and Zika viruses also entail human movement patterns^8,9,62,63^. Mosquito-borne diseases differ from droplet- and physical contact-transmission diseases because they require specific mosquito species and suitable environments for mosquito reproduction and virus development. Researchers must also consider intrinsic and extrinsic incubation periods between pairs of infected individuals. We believe that the EpiRank algorithm can be modified to make it useful for vector-borne disease studies.

There are other ways that EpiRank can be extended and modified for specific research requirements such as those involving trains, buses and rapid transit systems. To emphasize the effects of forward and backward movement, in the present study we simplified transportation mode differences by aggregating commuters between pairs of townships. In Taiwan, common commuting transportation modes include private vehicles (cars, motorcycles) and public transportation (buses, railways). In other countries they include ferries. Human interactions in different transportation types (e.g., crowded buses versus single-occupancy cars) result in different disease diffusion outcomes—in other words, different link types have various effects on infection processes^64^. EpiRank can be modified to consider a broad range of *FW* and *BW* patterns, with other weighting parameters used to integrate various transportation modes into calculations.

To assist in studies involving external factors, EpiRank provides an *exFac* term for integrating local environmental influences that have potential for altering location (node) vulnerability. The strength of the *exFac* effect on the disease spreading process is controlled by the damping factor—the lower the factor, the stronger the effect. Accordingly, *exFac* can be treated as the collective results of location-based socio-demographics or physical environmental factors such as prior infection history, population density, income level, daily temperature and precipitation, or air quality. We designed *exFac* to serve as a positive multiplier in equations so that it exerts a diffusion or increased susceptibility effect. When adding other external factors, researchers need to convert variables to ensure such positive effects. For example, if a higher population density leads to the greater likelihood of disease spread into a specific area, that means population density exerts a positive effect. In contrast, if a lower income level makes a location more vulnerable, that indicates a need to use an inverse income value for the *exFac* variable. If *exFac* can be modelled as a function of multiple variables, then collective model outcomes should be used with the EpiRank algorithm. Further, the addition of *exFac* means that the effects of a damping factor may express different patterns, thus requiring a damping factor sensitivity analysis.

Another possible EpiRank extension involves the spatial features of nodes. Since commuting networks are embedded in geographic spaces, distances between locations can wield strong influences on movement^14^. Our decision to not include the influences of distance in the version of EpiRank used in this study reflects the lack of certainty regarding the effects of distance on disease diffusion within a commuting network. More investigation is required to clarify the effects of distance on disease diffusion (e.g., the effect shapes of gravity, exponential, or radiation model functions) before adding this factor to EpiRank.

A third possible extension involves separating the influences of local flows. The term “local flow” refers to individuals who live and work in the same geographic location such as a township; it is expressed as a self-loop in the commuting network. In scenarios where diseases are spread among spatial network locations through inter-location links, one potential effect of local flow is strengthening nodes via larger populations. Since population size is another important factor influencing disease spreading mechanisms^65,66^, it should be modeled separately from and integrated into the link structure-based diffusion model.

There are several study limitations to be considered, and we will address three. First, the study did not distinguish between infection and spreading mode development processes. Different diseases have different incubation and latency periods, as well as different spreading mechanisms (e.g., through physical contact or via air, water, or a vector). These differences in epidemiology and etiology are key considerations when creating models. Second, location or land-use factors such as business, residential, agricultural or forestry status can affect infection patterns, as can terrain and prevailing weather systems. These factors were not included in this study because they require more analysis and experimentation with the EpiRank algorithm to capture their effects. Third, commuting network and disease data collection occurred at different times, the former in 2000 and the latter between 2000 and 2009. Since commuting information could not be extracted from the 2010 census due to changes in methodology, we used 2000 data as a substitute, thus requiring an assumption that commuting behaviors did not change dramatically during the period in-between.

## Data Availability

The processed data and the code for algorithm are available on the public repository: https://github.com/canslab1/EpiRank-Algorithm/.
